# Global achievement of maternal health-related sustainable development goals targets among women exposed to intimate partner violence

**DOI:** 10.1186/s12905-023-02577-9

**Published:** 2023-08-09

**Authors:** Amany Refaat

**Affiliations:** Faculty of Medicine, Public Health, Suez Canal University, Ismailia, Egypt

**Keywords:** Intimate Partyener Violence, Sustainable Development Goals, Maternal Health

## Abstract

**Abstract:**

Health-related Sustainable Development Goal (SDG 3) aims to ensure healthy lives. This study investigates the influence of Intimate Partner Violence (IPV) on achieving maternal health related SDG3 targets among exposed women globally.

**Methodology:**

This study used secondary data analysis of Domestic Violence Modules from the latest Demographic and Health Surveys (DHS) of 40 countries. IPV was defined by women ever exposed to emotional, physical, severe, or sexual violence from the spouse. Achieving maternal health related SDG3 targets among women was measured by: Proportion of births attended by skilled health personnel; Antenatal care (women received antenatal care eight or more times from any provider); and the need for family planning satisfied with modern methods. The influence of IPV was estimated through adjusted OR after controlling for socioeconomic factors using logistic regression.

**Results:**

Exposure to IPV was among one-third of the women (37%) mainly physical (29%) and emotional (24%). Adjusted OR with 95%CI for the influence of IPV on women’s utilization of skilled birth attendants was 0.81 (0.79–0.83)); having eight or more antenatal care visits 0.61 (0.59–0.63); and the needs for family planning with modern methods satisfied was 0.85 (0.83–0.87). Achievement of maternal health services was among 57% of the women. Women exposed to IPV were less to achieve maternal health services (50% VS 61%) with adjusted OR 0.71; 95%CI: 0.70–0.73 and it was not confounded by the positive effect of socioeconomic factors.

**Conclusion & Recommendation:**

Exposure to IPV had a statistically significant negative influence on all the maternal health related SDG3 services among women. Programs working in facilitating the achievement of the SDGs related maternal health targets should include prevention of women’s exposure to IPV and support for those who are exposed to it.

## Background

The Millennium Development Goal (MDGs) specifically MDG5, followed by the third UN’s Sustainable Development Goals (SDG3) aimed at improving health [1–6]. Maternal Health-related SDGs are SDG 3.1 aiming to reduce maternal mortality ratio and 3.7 aiming to ensure universal access to sexual and reproductive health-care services [7]. To achieve the maternal health-related SDGs, The World Health Organization (WHO) identified three main reproductive and maternal health services [8] as: Married or in-union women of reproductive age who need family planning satisfied with modern methods (%). The unmet need for family planning provides a measurement of the capacity of women to achieve their desired family size and birth spacing [9, 10]. Antenatal care coverage - at least four visits (%): The World Health Organization has issued a new series of recommendations to improve the quality of antenatal care to give women a positive pregnancy experience. The new guidance increases the number of contacts a pregnant woman has with health providers throughout her pregnancy from four to eight [11]. Births attended by skilled health personnel (%). Most maternal deaths are preventable with timely management by a skilled health professional (midwife, doctor, or nurse) working in a supportive environment [12, 13].

While the GBD 2017 study [14] found that maternal mortality ratio, and other SDG3 indicators had the most countries with at least 95% probability of target attainment; specific projection of some SDG 3 indicators [15] showed that neither of the maternal mortality ratio and the need for family planning satisfied with modern methods among other SDG3 indicators are on track to achieve their respective SDG targets by 2030. Different challenges in achieving health related SDG were mentioned and innovative approaches of political commitment and financial mobilization were endorsed to face them [16, 17].

There is no shortage in the body of literature about the socioeconomic determinants in achieving these reproductive health services in low- and middle-income countries [18–26].

On the other hand, Intimate Partner Violence (IPV) is a prevalent worldwide health problem. IPV is underreported and underrecognized by healthcare professionals. IPV causes significant physical and psychiatric health problems with more victimization among women, especially in low- and middle-income countries [27, 28].

There is a plethora of evidence-based studies on the vulnerabilities of women exposed to IPV to inadequate utilization of maternal health care services and adverse maternal health outcomes [29–35]. However, there is a gap of literature investigating the influence of women exposure to IPV on their achievement of combined maternal health services as related to SDGs Targets.

Therefore, the present study will focuse on the influence of Intimate Partner Violence (IPV) on it. It will investigate IPV’s influence on women’s utilization of these three reproductive health services.

## Methodology

### Study design

This study used secondary data analysis of Domestic Violence Modules of Demographic and Health Surveys (DHS), which are nationally representative surveys that employ standardized questionnaires to collect extensive data from women of reproductive age (15–49 years) in developing countries. The DHS obtain information on women’s sociodemographic characteristics, their reproductive behaviors, birth history and maternal health service utilization [36].

### Datasets

DHS collected data on women’s exposure to emotional, physical, and sexual violence for over 25 countries and growing [37] using a domestic violence module [38]. Individual Women’s Data – Individual Recode (IR) files of DHS have one record for every eligible woman as defined by the household schedule. It contains all the data collected in the women’s questionnaire plus some variables from the household. The unit of analysis (case) in this file is the woman [39]. In DHS surveys, weights need to be applied when tabulations are made of statistics to produce the proper representation. When weights are calculated because of sample design, corrections for differential response rates are also made [40]. Weights were computed by dividing the sampling weighting variable (v005) by 1,000,000 and accounting for stratification and clustering.

Datasets from the standard DHS of 40 countries that collected domestic violence module using DHS questionnaire version VII [41] starting from 2014 were downloaded in SPSS format and included in this study.

### Population

The 40 countries were from different economic levels as the following according to the most recent World Bank economic GNI Per Capita classification [42].:

#### Low-income economics

(GNI per Capita $1,045 OR LESS):

Afghanistan (2015); Burundi (2016); Chad (2014); Congo Democratic Republic (2014); Ethiopia (2016); Gambia (2019); Liberia (2019); Malawi (2015); Mali (2018); Mozambique (2015); Nepal (2016); Rwanda (2015); Sierra Leone (2019); Tanzania (2016); Togo (2014); and Uganda (2016).

#### Lower-middle income economics

(GNI per Capita $1,046 TO $4,095):

Angola (2015); Benin (2017); Cameroon (2018); Cambodia (2014); Egypt (2014); Haiti (2016); India (2019); Kenya (2014); Mauritania (2019); Myanmar (2016); Nigeria (2018); Pakistan (2017); Papua New Guinea (2018); Philippines (2017); Tajikistan (2017); Timor-Leste (2016); Zambia (2018); and Zimbabwe (2015).

#### Upper-middle income economics

(GNI per Capita $4,096 TO $12,695):

Armenia (2016); Colombia (2015); Guatemala (2015); Jordan (2017); Maldives (2016); and South Africa (2016).

Geographically, they presented countries from the following DHS regions [43]: Sub-Saharan Africa; North Africa/West Asia/Europe; Central Asia; South & Southeast Asia, and Latin America & Caribbean.

### Variables

#### Outcome variable

Achievement of maternal health-related SDG targets were measured through three main reproductive and maternal health services as the following:

##### The proportion of married or in-union women of reproductive age who have their need for family planning satisfied with modern methods:

It was coded from the revised DHS variable of unmet needs for family planning (variable 626 A). The unmet need for family planning is defined as the percentage of women who do not want to become pregnant but are not using contraception. Though the concept seems straightforward, the calculation is extraordinarily complex and has changed over time. To address these issues, DHS revised the definition of unmet need in 2012 [44].

##### Antenatal care coverage:

Having adequate antenatal care visits number was coded from the variable of number of antenatal care visits to be 8 and more visits for the last child according to the new guidelines of WHO [11].

##### Births attended by skilled health personnel (%):

Skilled birth attendant was computed from assisted delivery by a doctor or nurse/midwife for the last birth.

A new variable was counted from the three maternal health services. It was dichotomized to those who achieved two or the entire three versus those who were not achieving any or just one of the three.

### Predictor variables

#### Intimate partner violence

Exposure of Women to Intimate Partner Violence (IPV) was measured by counting the answers of yes for the four types of violence [45]. A new variable of IPV was coded from women exposure to at least one type.

Emotional violence was measured from the answer of yes to “Experienced any emotional violence” which counts the answers of variables of “Ever humiliated by husband/partner”; “Ever threatened with harm by husband/ partner” and “Ever insulted or made her feel bad by husband/ partner”.

Physical violence was measured by answer of yes to question. “Experienced any less severe violence” which counts the answers of from variables of “Ever been pushed, shook, or have something thrown by husband/partner”, “Ever been slapped by husband/partner”, “Punched with fist, or hit by something harmful by husband/partner”, and “Ever had arm twisted or hair pulled by husband/partner”.

Severe physical violence was measured by answer of yes to question “Experienced any severe violence” which counts answers of “Ever been kicked or dragged by husband/partner”, “Ever been strangled or burnt by husband/partner” and “Ever been threatened with a knife /gun or other weapon by husband/partner”.

Sexual violence was by the answer of yes to “Experienced any sexual violence” which counts the answers of “Ever been physically forced into unwanted sex by husband/partner”, “Ever been forced into other unwanted sexual acts by husband/partner” and “Ever been physically forced to perform sexual acts respondent didn’t want to by husband/partner”.

#### Socioeconomic determinants

The socioeconomic determinants of exposure to IPV and utilization of reproductive health services were counted from the following variables:

Women age: it was measured through the variable” Women age in 5 years groups” classified as ages 15–19; 20–24; 25–29; 30–34; 35–39; 40–44; and 45–49 years old.

Residence: it was measured from variable “Type of place of residence” of Urban Vs. Rural residence.

Economic level of household: It was measured from the “Wealth index” variable of DHS data categorizing economic level into five levels of poorest, poor, middle, richer, and richest.

Women’s Educational level: it was measured from the variable of “Highest educational level” of no education, primary, secondary, and higher.

Women ‘s working status: The working status variable was computed from the answer of yes to variable of “Respondent is currently working”.

Economic level of the country: it was measure through the World Bank countries classification.

A scale of the socioeconomic determinants was computed from these variables. It was recoded as low, middle, and high levels.

### Data analysis plan

Data analysis using SPSS version 24 was conducted, including descriptive statistics as frequencies and significance tests (Odd Ratio with 95%Confidence Intervals). The valid number of observations was counted excluding those with answers of “Don’t know” to tackle the well-known limitations of the cross-sectional study nature of DHS and recall bias.

The StatCalc function of Epi-info software was used to estimate Chi-Square for trend and related OR, 95%CI. The influence of IPV and socioeconomic level were measured through running significance tests. The influence of each type of IPV on maternal health related SDGs targets was estimated through significance tests. The influence of IPV was tested with OR, 95% CI on a newly coded variable of the utilization of maternal health services among different countries. To identify that the influence of IPV on the achievement of maternal health related SDGs target is not confounded by the socioeconomic level, logistic regression analysis was conducted.

## Results

The final weighted data was from 290,825 women who responded to the domestic module questionnaire. Exposure to emotional violence was among 24% of women; however, reached 50% in Papua New Guinea, followed by Sierra Leon (46%). Less severe physical violence was among 29%; however, it was among 52% in Papua New Guinea, followed by Afghanistan (50%). Severe violence was experienced by 11% of women, it was highest in Papua New Guinea (35%) followed by Sierra Leon (29%). The exposure to sexual violence was among 9% of women, reaching 28% in Papua New Guinea, followed by Burundi (26%).

IPV was among 37% of women mostly as one type (17%) while only 3% were exposed to the whole 4 types of violence. The most prevalent violent behaviors were slapping (25%), followed by insulting (18%), and pushing (16%). While the least were burning, strangling, or threatening by gun/knife (less than 3% each).

**Fig. 1:**
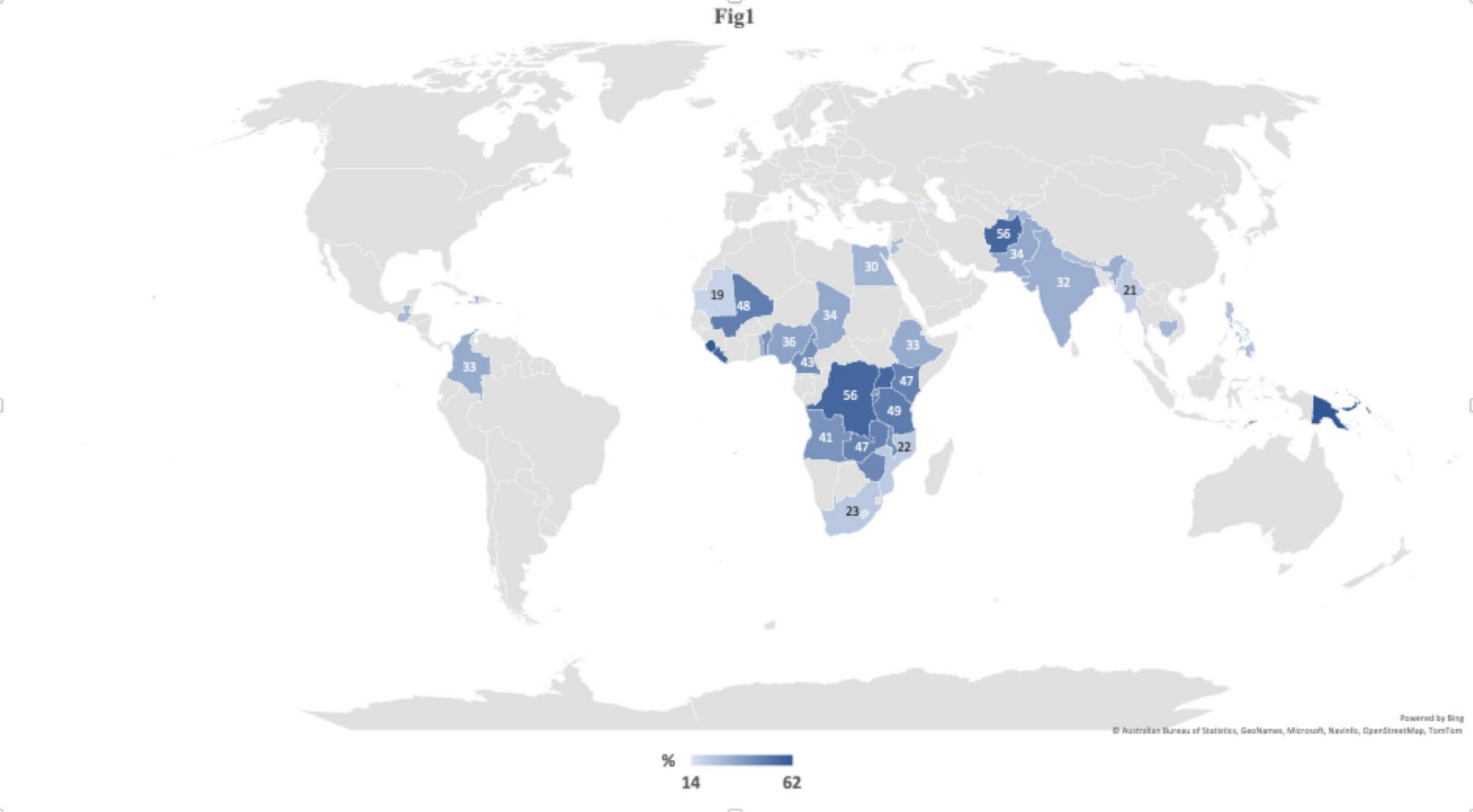
Prevalence of intimate partner violence

The prevalence of IPV among countries as demonstrated in Fig. 1 showed that it was highest in Papua New Guinea (62%), followed by Sierra Leon (61%) and it was among 56% in Afghanistan, Liberia, and Uganda. While it was lowest in Armenia (14%) and Mauritania (19%).

### Socioeconomic determinants

**Fig. 2:**
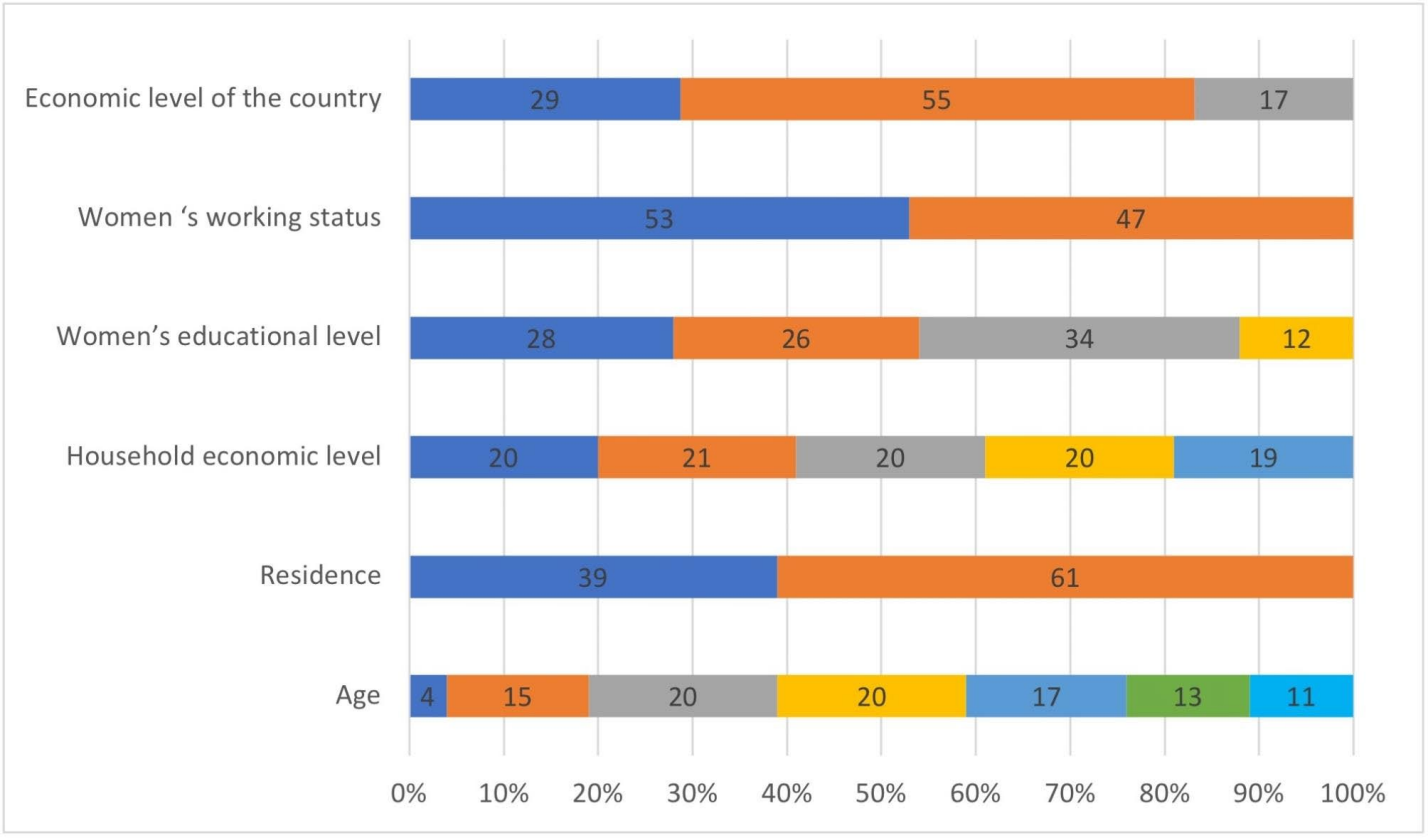
Socioeconomic characteristics of women

As Fig. 2 shows, the highest percentage of the women in the study population were at age of 25–34 years old (40%), while young women of 15–19 years old constituted 4% only. Most of the women were residing in rural areas (61%); however, 91% of women in Burundi and Papua New Guinea lived in rural areas and only 10% in Jordan. They were almost equally distributed among the five levels of household income in all countries. More than half of women resided in low medium income countries.

More than half of the women were either uneducated (28%) or educated to basic level (26%), while one third (34%) were educated to the high school level. Highly educated women constituted 12% only. Highest number of uneducated women was in Afghanistan (83%), followed by Mali (72%).

More than half of the women (53%) were not currently working. Working women were highest in Burundi (88%), Rwanda (87%) and Benin (85%); while they were the least in Afghanistan (12%). More than half of the women were self-employed (52%), highest in Congo (89%) and Haiti (86%). While 28% of women were working for family, highest in Jordan (93%). Highest working type was self-employed agricultural (27%), reaching up to 89% of working women in Burundi, followed by working in sales (21%), highest in Haiti (75%).

### Socioeconomic scale

The socioeconomic scale ranged from 6 to 21 with median of 12. It was recoded to low (less than 10); middle (11–15) and high (16+) levels. Most of the participants were of middle level (60%), while those in low level constituted 26% and 14% were in the high level. The socioeconomic scale was dichotomized to low and higher levels (middle and high) which constituted 74%.

Achieving Maternal Health-related SDGs targets among women:

### Proportion of married or in-union women of reproductive age who have their need for family planning satisfied with modern methods

Unmet need for contraception, among women currently in union was among 16% of 261,142 women. Thus 84% of women had met needs of modern contraception. The highest unmet need was in Angola and Haiti (38% each) followed by Togo (34%) and Liberia (33%). Met needs was highest in Colombia (93%), India (91%) and Zimbabwe (90%).

As Table 1 shows. Met need for family planning was less among women exposed to IPV (82% Vs 85%) with OR 0.80; 95%CI: 0,78-0.82). Higher socioeconomic level was a positive determinant for met needs of family planning methods (OR 1.74; 95%CI: 1.70–1.77).

### Antenatal care coverage

Valid results from 155,123 women showed that 11% of them didn’t not have antenatal care visits at all during their last pregnancy and the median and mode among others was 4 visits. Having adequate antenatal care visits number for at least 8 antenatal visits for the last child was among 16% only of women,

Having 8 or more visits was highest in the Maldives (83%), Jordan (73%) and Egypt (58%). While it was less than 1% at Burindi, Rwanda, and Chad.

As Table 1 shows, having 8 or more antenatal care visits was less among those exposed to IPV (OR: 0.55; 95%CI: 0. 54-0.57). Higher socioeconomic level had three times positive impact on having antenatal care visits (OR 3.59; 95%CI:3.47–3.72).

### Delivery assisted by skilled birth attendants

Valid responses from 154,524 women, showed that Skilled birth attendants (SBA) assisted in 72% of deliveries (mainly as nurses/midwives 44%). Traditional birth attendants (TBA) assisted in 15% of deliveries, while about 12% of deliveries were assisted by others as relatives or friends.

Delivery assisted by skilled birth attendants was universal (100%) in Armenia, Jordan, and the Maldives, all high middle-income countries. While it was lowest in Tanzania (13%), Chad (27%) and Ethiopia (30%).

As Table 1 shows. deliveries assisted by skilled health attendants were less among women exposed to IPV ( OR 0.73; 95%CI:0.71–0.74)). Higher socioeconomic level was a three times positive determinant for having assisted deliveries through skilled birth attendants (OR 2.83; 95%CI: 2.76–2.89).

**Table 1:** Influence of IPV exposure on achieving health-related SDG targets (OR, 95%CI)

Variable	Met need of contraception	Having eight or more antenatal care visits	Delivery assisted by skilled health personnel
Exposure to IPV	0.80 (0.78–0.82	0.55 (0.54–0.57)	0.73 (0.71–0.74)
Higher socioeconomic level	1.74 (1.70–1.77)	3.59 (3.47–3.72)	2.83 (2.76–2.89)

The results showed that IPV had a negative influence on each of the three maternal health service; however, having proper antenatal care was the most affected one. Higher socioeconomic levels increased their utilization.

### Achievement of maternal health related SDGs targets

The new variable of achievement of maternal health services showed that 12% only utilized the three services, 45% utilized two of the three, and 33% utilized one only, while 10% did not use any of them.

**Fig. 3:**
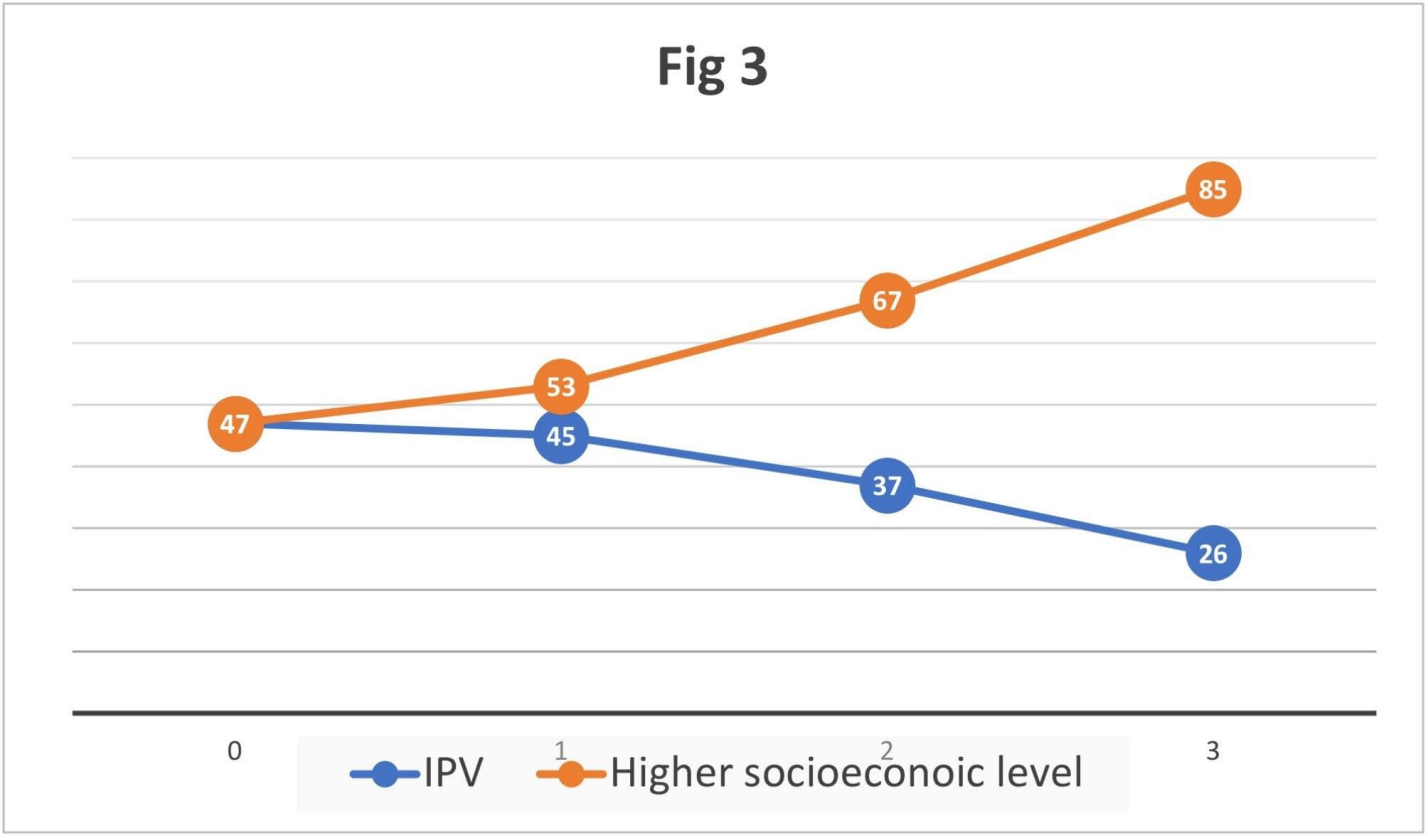
Influence of both exposure to IPV and higher socioeconomic levels on the number of maternal health related services

As Fig. 3 shows, exposure to IPV was higher with less or no services in contrast to higher socioeconomic level that increases services number.

Most of the women (57%) utilized maternal health services (at least two of the three services), while the rest 43% were considered not utilizing. Achievement of the maternal health related SDGs was highest in Jordan (93%), Maldives (90%), India (89%) then Egypt (87%), while it was the least in Tanzania (10%).

As Table 2 shows all types of IPV influenced negatively the three services and the total achievement of maternal health related SDGs targets. The strongest influence was from exposure to severe physical violence and sexual violence.

**Table 2:** Influence of different types of IPV on the achievement of maternal health related SDGs (OR; 95%CI)

	Met need of contraception	Having eight or more antenatal care visits	Delivery assisted by skilled health personnel	Achievement of maternal health related SDGs
Emotional violence	0.74 (0.72–0.76)	0.63 (0.61–0.65)	0.73 (0.72–0.75)	0.65 (0.64–0.67)
Physical violence	0.88 (86 − 0.90)	0.55 (0.53–0.57)	0.72 (0.70–0.74)	0.67 (0.66–0.69)
Severe physical violence	0.78 (0.75–0.81)	0.56 (0.53–0.59)	0.70 (0.68–0.73)	0.58 (0.56–0.60)
Sexual violence	0.68 (0.66–0.71)	0.41 (0.39–0.44)	0.80 (0.77–0.83)	0.61(0.59–0.63)

Women exposed to IPV were less to achieve maternal health services (OR 0.66; 95%CI: 0.64–0.67).

**Fig .4:**
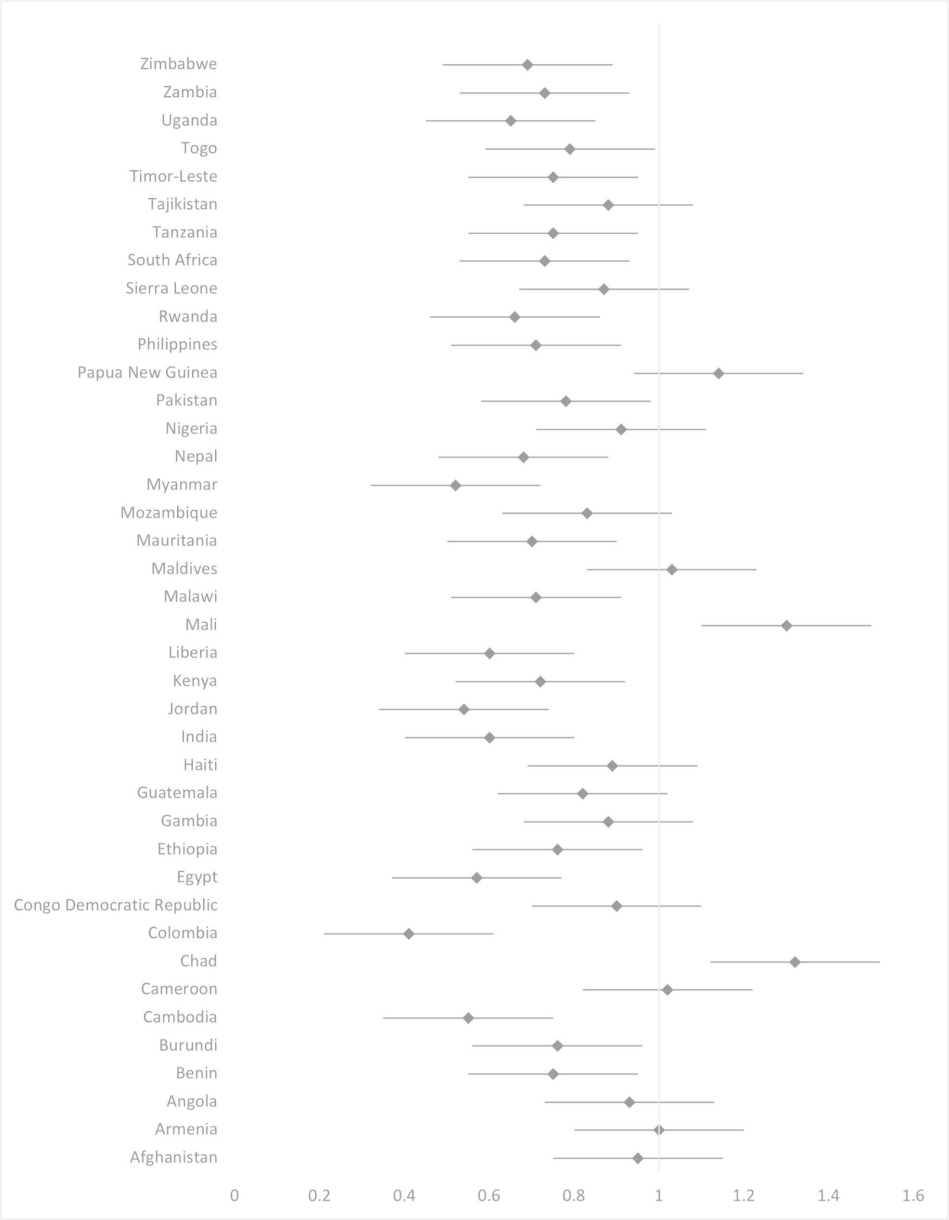
Influence of IPV on achievement of maternal health services among countries

The negative influence of IPV on the achievement of maternal health services was in almost all countries; however, the strongest influence was in Colombia (OR 0.41; 95%CI: 0.37–0.46) then Myanmar (OR: 0.52; 95%CI: 0.40–0.66). (Fig. 4). While, it has a positive influence in Chad (OR: 1.32; 95%CI: 1.10–1.61) and Mali (OR: 1.30; 95%CI: 1.11–1.53).

As Table 3 shows, the negative effect of exposure to IPV did not lose its significance and was not confounded by the double positive effect of higher socioeconomic level.

**Table 3:** Influence of IPV on the achievement of maternal health related SDGs (OR 95% CI of Logistic regression analysis

	Model 1	Model 2
IPV	0.66 (0.64–0.67)	0.68 (0.67–0.70)
Higher socioeconomic level		2.23(2.18–2.28)
Constant	0.44	-0.87
Model significance	0.000	0.000

## Discussion

Despite the wealth of literature that investigated the influence of different types of violence on maternal health, there was a need to identify this influence on the combined maternal health related SDGs. The present study found that IPV prevalence was among 37% of the women like the global estimates of 1 in 3 (35%) by the WHO [46]. Papua New Guinea which has the highest prevalence of IPV (62%) is a low middle income country with GNI per capita $2,790 [47], maternal mortality ratio of 199 per 100,000 [48] at the same year data collection (2018), and a GINI Index of 41.9 [49]. The Gini Index is a summary measure of income inequality [50]. A higher Gini index indicates greater inequality, with high-income individuals receiving much larger percentages of the population’s total income [51]. Further analysis of its socioeconomic levels showed that most if its population (63%) live in the middle level and 27% in the lower level with 10% only in the higher level. However, 91% of its population reside in rural areas, and 78% of its women are either not educated (30%) or have primary level of education (48%). While Sierra Leon is a low-income country with GNI per Capita $ 510 and GINI Index of 35.7 [52]. The majority of its population are living in the lower (41%) and middle (57%) socioeconomic levels. Most of its population (65%) live in rural areas and most of its women (60%) are uneducated. This could shed light on the influence of the socioeconomic conditions in these two countries having the highest levels of IPV.

Colombia is a high middle-income country with GNI per capita ($6,160) and GINI index 54.2 [53]. It has a high achievement of maternal health services (83%); however, women exposed to IPV (33%) have lower level of 74% only. While Myanmar is a low-middle income country with GNI per Capita $1,140 and GINI Index of 30.7 [54]. It has achievement of maternal health services of 58%; less among those exposed to IPV (45%) despite that IPV is among 21% of its women.

Surprisingly, IPV had a positive effect on achievement of maternal health services in two countries: Chad (OR 1.32; 95% CI: 1.08–1.61) and Mali (1.30; 95%CI: 1.13–1.53). Both countries are low-income countries. Chad has GNI per Capita $650 and GINI Index of 37.5 [55]. It has IPV prevalence of 33% and achievement of maternal health among 17% only which rises to 20% among those exposed to IPV. Its women were universally living in lower (54%) and middle (45%) socioeconomic level; mostly in rural residence (79%) and in their majority they were either not educated (68%) or with primary level of education (21%). Those women educated to primary level were the highest to be exposed to IPV (44%); however, they were not the highest to achieve Maternal health services. On the other hand, Mali has a GNI per capita of $870 and a GINI index of 36.1 [56]. Almost half of its women (49%) achieve maternal health services which is increased to 52% among those exposed to IPV. Women were universally living in low (51%) or middle (48%) socioeconomic levels. Most of the women live in rural areas (79%) and are uneducated (72%) or have a primary level of education (13%). Those with primary education were the highest to be exposed to IPV (54%); however, they were not the highest to achieve Maternal health services. The available data does not explain this positive influence of IPV on the achievement of maternal health services in these two countries and that calls for further study.

## Conclusion & recommendation

Globally, one-third of women are exposed to Intimate Partner Violence (IPV). IPV influenced their achievement of Maternal health-related SDGs targets negatively as it lowered their utilization of Skilled Birth Attendants, having at least eight ante-natal care visits, and having their met needs of modern contraceptive methods. The influence was statistically significant and not confounded by the socioeconomic factors. Programs working in facilitating the achievement of the SDGs related maternal health targets should include prevention of women’s exposure to IPV and support for those who are exposed to it. The study recommends introduction of prevention of IPV in health professional educational curricula to train them to be more responsive to the health needs of exposed women and more competent in achieving the maternal health related SDGs.

## Data Availability

The data that support the findings of this study are available from DHS website, but restrictions apply to the availability of these data, which were used under approval for the current study, and so are not to be shared by the author. The datasets in SPSS format were downloaded from DHS website for the following countries: Afghanistan (2015): https://dhsprogram.com/data/dataset/Afghanistan_Standard-DHS_2015.cfm?flag=0. Angola (2015): https://dhsprogram.com/data/dataset/Angola_Standard-DHS_2015.cfm?flag=1. Armenia (2016): https://dhsprogram.com/data/dataset/Armenia_Standard-DHS_2016.cfm?flag=1. Benin (2017): https://dhsprogram.com/data/dataset/Benin_Standard-DHS_2017.cfm?flag=1. Burundi (2016): https://dhsprogram.com/data/dataset/Burundi_Standard-DHS_2016.cfm?flag=1. Cambodia (2014): https://dhsprogram.com/data/dataset/Cambodia_Standard-DHS_2014.cfm?flag=1. Cameroon (2018): https://dhsprogram.com/data/dataset/Cameroon_Standard-DHS_2018.cfm?flag=1. Chad (2014): https://dhsprogram.com/data/dataset/Chad_Standard-DHS_2014.cfm?flag=1. Colombia (2015): https://dhsprogram.com/data/dataset/Colombia_Standard-DHS_2015.cfm?flag=1. Congo Democratic Republic (2014): https://dhsprogram.com/data/dataset/Congo-Democratic-Republic_Standard-DHS_2013.cfm?flag=1. Egypt (2014): https://dhsprogram.com/data/dataset/Egypt_Standard-DHS_2014.cfm?flag=1. Ethiopia (2016): https://dhsprogram.com/data/dataset/Ethiopia_Standard-DHS_2016.cfm?flag=1. Gambia (2019): https://dhsprogram.com/data/dataset/Gambia_Standard-DHS_2019.cfm?flag=1. Guatemala (2015): https://dhsprogram.com/data/dataset/Guatemala_Standard-DHS_2015.cfm?flag=1. Haiti (2016): https://dhsprogram.com/data/dataset/Haiti_Standard-DHS_2016.cfm?flag=1. India (2019): https://dhsprogram.com/data/dataset/India_Standard-DHS_2020.cfm?flag=1. Jordan (2017): https://dhsprogram.com/data/dataset/Jordan_Standard-DHS_2017.cfm?flag=1. Kenya (2014): https://dhsprogram.com/data/dataset/Kenya_Standard-DHS_2014.cfm?flag=1. Liberia (2019): https://dhsprogram.com/data/dataset/Liberia_Standard-DHS_2019.cfm?flag=1. Malawi (2015): https://dhsprogram.com/data/dataset/Malawi_Standard-DHS_2015.cfm?flag=1. Maldives (2016): https://dhsprogram.com/data/dataset/Maldives_Standard-DHS_2016.cfm?flag=1. Mali (2018): https://dhsprogram.com/data/dataset/Mali_Standard-DHS_2018.cfm?flag=1. Mauritania (2019): https://dhsprogram.com/data/dataset/Mauritania_Standard-DHS_2020.cfm?flag=1. Mozambique (2015): https://dhsprogram.com/data/dataset/Mozambique_Standard-AIS_2015.cfm?flag=1. Myanmar (2016): https://dhsprogram.com/data/dataset/Myanmar_Standard-DHS_2016.cfm?flag=1. Nepal (2016): https://dhsprogram.com/data/dataset/Nepal_Standard-DHS_2016.cfm?flag=1. Nigeria (2018): https://dhsprogram.com/data/dataset/Nigeria_Standard-DHS_2018.cfm?flag=1. Pakistan (2017): https://dhsprogram.com/data/dataset/Pakistan_Standard-DHS_2017.cfm?flag=1. Papua New Guinea (2017): https://dhsprogram.com/data/dataset/Papua-New-Guinea_Standard-DHS_2017.cfm?flag=1. Philippines (2017): https://dhsprogram.com/data/dataset/Philippines_Standard-DHS_2017.cfm?flag=1. Rwanda (2015): https://dhsprogram.com/data/dataset/Rwanda_Standard-DHS_2015.cfm?flag=1. Sierra Leone (2019): https://dhsprogram.com/data/dataset/Sierra-Leone_Standard-DHS_2019.cfm?flag=1. South Africa (2016): https://dhsprogram.com/data/dataset/South-Africa_Standard-DHS_2016.cfm?flag=1. Tajikistan (2017): https://dhsprogram.com/data/dataset/Tajikistan_Standard-DHS_2017.cfm?flag=1. Tanzania (2016): https://dhsprogram.com/data/dataset/Tanzania_Standard-DHS_2015.cfm?flag=1. Timor-Leste (2016): https://dhsprogram.com/data/dataset/Timor-Leste_Standard-DHS_2016.cfm?flag=1. Togo (2014): https://dhsprogram.com/data/dataset/Togo_Standard-DHS_2013.cfm?flag=1. Uganda (2016): https://dhsprogram.com/data/dataset/Uganda_Standard-DHS_2016.cfm?flag=1. Zambia (2018): https://dhsprogram.com/data/dataset/Zambia_Standard-DHS_2018.cfm?flag=0. Zimbabwe (2015): https://dhsprogram.com/data/dataset/Zimbabwe_Standard-DHS_2015.cfm?flag=1.

## References

[1] WHO | MDG 5: improve maternal health [Internet]. WHO. World Health Organization; [cited 2020 Apr 5]. Available from: https://www.who.int/topics/millennium_development_goals/maternal_health/en/.

[2] WHO | Millennium Development Goals 4 and 5 [Internet]. WHO. World Health Organization; [cited 2020 May 4]. Available from: https://www.who.int/pmnch/about/about_mdgs/en/.

[3] Transforming our world: the 2030 Agenda for Sustainable Development. Sustainable Development Knowledge Platform [Internet]. [cited 2020 May 4]. Available from: https://sustainabledevelopment.un.org/post2015/transformingourworld.

[4] Voss M, Marten R, Gulati D. Accelerating the SDG3 Global Action Plan. BMJ Global Health [Internet]. 2019 Sep 1 [cited 2019 Dec 27];4(5). Available from: https://gh.bmj.com/content/4/5/e001930. 10.1136/bmjgh-2019-001930 PMC6747908 31565423

[5] GBD 2016 SDG Collaborators (2017). Measuring progress and projecting attainment on the basis of past trends of the health-related Sustainable Development Goals in 188 countries: an analysis from the global burden of Disease Study 2016. Lancet.

[6] WHO | SDG 3: Ensure healthy lives and promote wellbeing for all at all ages [Internet]. WHO. World Health Organization; [cited 2020 May 4]. Available from: http://www.who.int/sdg/targets/en/.

[7] SDG Indicators — SDG Indicators [Internet]. [cited 2020 May 4]. Available from: https://unstats.un.org/sdgs/metadata/.

[8] Maternal and reproductive health [Internet]. [cited 2022 May 20]. Available from: https://www.who.int/data/gho/data/themes/topics/topic-details/GHO/maternal-and-reproductive-health.

[9] Wulifan JK, Jahn A, Hien H, Ilboudo PC, Meda N, Robyn PJ (2017). Determinants of unmet need for family planning in rural Burkina Faso: a multilevel logistic regression analysis. BMC Pregnancy Childbirth.

[10] Kantorová V, Wheldon MC, Ueffing P, Dasgupta ANZ (2020). Estimating progress towards meeting women’s contraceptive needs in 185 countries: a bayesian hierarchical modelling study. PLOS Med.

[11] New guidelines on antenatal care for a positive pregnancy experience. Accessed May 8, 2021. https://www.who.int/news/item/07-11-2016-new-guidelines-on-antenatal-care-for-a-positive-pregnancy-experience.

[12] Maternal mortality. Accessed May 10, 2021. https://www.who.int/news-room/fact-sheets/detail/maternal-mortality.

[13] WHO | Definition of skilled health. personnel providing care during childbirth: WHO-RHR-18.14-eng.pdf [Internet]. [cited 2022 May 20]. Available from: https://apps.who.int/iris/bitstream/handle/10665/272818/WHO-RHR-18.14-eng.pdf.

[14] Lozano (2018).

[15] Strong K, Noor A, Aponte J, Banerjee A, Cibulskis R, Diaz T, Ghys P, Glaziou P, Hereward M, Hug L, Kantorova V, Mahy M, Moller A-B, Requejo J, Riley L, Say L, You D (2020). Monitoring the status of selected health related sustainable development goals: methods and projections to 2030. Glob Health Action.

[16] Siddiqi S, Aftab W, Siddiqui FJ, Huicho L, Mogilevskii R, Friberg P, Lindgren-Garcia J, Causevic S, Khamis A, Shah MM, Bhutta ZA (2020). Global strategies and local implementation of health and health-related SDGs: lessons from consultation in countries across five regions. BMJ Glob Health.

[17] Aftab W, Siddiqui FJ, Tasic H, Perveen S, Siddiqi S, Bhutta ZA (2020). Implementation of health and health-related sustainable development goals: progress, challenges and opportunities – a systematic literature review. BMJ Glob Health.

[18] Haileamlak A (2018). Maternal and newborn mortality- still the Greatest disparity between low-income and high-income countries. Ethiop J Health Sci.

[19] Haider MR, Rahman MM, Moinuddin M, Rahman AE, Ahmed S, Khan MM (2017). Impact of maternal and neonatal health initiatives on inequity in maternal health care utilization in Bangladesh. PLoS ONE.

[20] Li Y, Zhang Y, Fang S, Liu S, Liu X, Li M (2017). Analysis of inequality in maternal and child health outcomes and mortality from 2000 to 2013 in China. Int J Equity Health.

[21] Harris DE, Aboueissa A-M, Baugh N, Sarton C (2015). Impact of rurality on maternal and infant health indicators and outcomes in Maine. Rural Remote Health.

[22] Awasthi A, Pandey CM, Chauhan RK, Singh U (2016). Disparity in maternal, newborn and child health services in high focus states in India: a district-level cross-sectional analysis. BMJ Open.

[23] De Groot A, Van de Munt L, Boateng D, Savitri AI, Antwi E, Bolten N (2019). Equity in maternal health outcomes in a middle-income urban setting: a cohort study. Reproductive Health.

[24] Makate M, Makate C (2017). The evolution of socioeconomic status-related inequalities in maternal health care utilization: evidence from Zimbabwe, 1994–2011. Glob Health Res Policy.

[25] Alosaimi AN, Nwaru B, Luoto R, Al Serouri AW, Mouniri H (2019). Using Household Socioeconomic Indicators to predict the utilization of maternal and Child Health Services among Reproductive-Aged women in rural Yemen. Glob Pediatr Health.

[26] Wuneh AD, Medhanyie AA, Bezabih AM, Persson L, Schellenberg J, Okwaraji YB (2019). Wealth-based equity in maternal, neonatal, and child health services utilization: a cross-sectional study from Ethiopia. Int J Equity Health.

[27] Dicola D, Spaar E (2016). Intim Partn Violence AFP.

[28] Ahmadabadi Z, Najman JM, Williams GM, Clavarino AM, d’Abbs P (2021). Gender differences in intimate Partner Violence in Current and Prior Relationships. J Interpers Violence.

[29] Mohammed BH, Johnston JM, Harwell JI, Yi H, Tsang KW-K, Haidar JA (2017). Intimate partner violence and utilization of maternal health care services in Addis Ababa, Ethiopia. BMC Health Serv Res.

[30] Musa A, Chojenta C, Geleto A, Loxton D (2019). The associations between intimate partner violence and maternal health care service utilization: a systematic review and meta-analysis. BMC Women’s Health.

[31] Yaya S, Gunawardena N, Bishwajit G (2019). Association between intimate partner violence and utilization of facility delivery services in Nigeria: a propensity score matching analysis. BMC Public Health.

[32] Metheny N, Stephenson R (2017). Intimate Partner Violence and Uptake of Antenatal Care: a scoping review of low- and Middle-Income Country Studies. Int Perspect Sex Reprod Health.

[33] Chaves K, Eastwood J, Ogbo FA, Hendry A, Jalaludin B, Khanlari S (2019). Intimate partner violence identified through routine antenatal screening and maternal and perinatal health outcomes. BMC Pregnancy Childbirth.

[34] Dhar D, McDougal L, Hay K, Atmavilas Y, Silverman J, Triplett D (2018). Associations between intimate partner violence and reproductive and maternal health outcomes in Bihar, India: a cross-sectional study. Reprod Health.

[35] Shamu S, Munjanja S, Zarowsky C, Shamu P, Temmerman M, Abrahams N (2018). Intimate partner violence, forced first sex and adverse pregnancy outcomes in a sample of zimbabwean women accessing maternal and child health care. BMC Public Health.

[36] The DHS Program - Data [Internet]. [cited 2020 Jan 3]. Available from: https://dhsprogram.com/Data/.

[37] The DHS Program - Gender Corner Main Page [Internet]. [cited 2022 May 18]. Available from: https://dhsprogram.com/topics/gender-corner/.

[38] DHS6_Module_Domestic_Violence_6Aug2014_DHSQMP. pdf [Internet]. [cited 2022 May 18]. Available from: https://dhsprogram.com/pubs/pdf/DHSQMP/DHS6_Module_Domestic_Violence_6Aug2014_DHSQMP.pdf.

[39] The DHS Program - Dataset Types [Internet]. [cited 2022 Apr 18]. Available from: https://dhsprogram.com/data/Dataset-Types.cfm.

[40] Guide to DHS Statistics [Internet]. [cited 2022 Apr 18]. Available from: https://dhsprogram.com/Data/Guide-to-DHS-Statistics/index.cfm.

[41] DHS Model. Questionnaire - Phase 7 (English, French) [Internet]. [cited 2022 May 18]. Available from: https://dhsprogram.com/publications/publication-dhsq7-dhs-questionnaires-and-manuals.cfm.

[42] World Bank Country and Lending Groups. – World Bank Data Help Desk [Internet]. [cited 2022 Apr 18]. Available from: https://datahelpdesk.worldbank.org/knowledgebase/articles/906519-world-bank-country-and-lending-groups.

[43] The DHS Program - Country List [Internet]. [cited 2022 May 8]. Available from: https://dhsprogram.com/Countries/Country-List.cfm.

[44] The DHS Program - Research Topics - Unmet Need for Family Planning [WWW Document], n.d. URL https://dhsprogram.com/topics/Unmet-Need.cfm (accessed 9.8.22).

[45] Frequently asked questions: Types of violence against women and girls [Internet]. UN Women – Headquarters. [cited 2022 May 11]. Available from: https://www.unwomen.org/en/what-we-do/ending-violence-against-women/faqs/types-of-violence.

[46] Violence against women [WWW Document]., n.d. URL https://www.who.int/health-topics/violence-against-women (accessed 6.14.23).

[47] GNI per capita., Atlas method (current US$) - Papua New Guinea | Data [WWW Document], n.d. URL https://data.worldbank.org/indicator/NY.GNP.PCAP.CD?locations=PG (accessed 9.30.22).

[48] Maternal mortality ratio. (per 100 000 live births) [WWW Document], n.d. URL https://www.who.int/data/gho/data/indicators/indicator-details/GHO/maternal-mortality-ratio-(per-100-000-live-births) (accessed 6.14.23).

[49] Gini index -. Papua New Guinea | Data [WWW Document], n.d. URL https://data.worldbank.org/indicator/SI.POV.GINI?locations=PG (accessed 9.30.22).

[50] Bureau UC. n.d. Gini Index [WWW Document]. Census.gov. URL https://www.census.gov/topics/income-poverty/income-inequality/about/metrics/gini-index.html (accessed 9.30.22).

[51] Gini Index Explained and Gini Coefficients Around the World [WWW, Document], n.d., Investopedia. URL https://www.investopedia.com/terms/g/gini-index.asp (accessed 9.30.22).

[52] Gini index - Sierra Leone | Data [WWW Document], n.d. URL https://data.worldbank.org/indicator/SI.POV.GINI?locations=SL (accessed 9.30.22).

[53] Gini index - Colombia. | Data [WWW Document], n.d. URL https://data.worldbank.org/indicator/SI.POV.GINI?locations=CO (accessed 9.30.22).

[54] Gini index - Myanmar. | Data [WWW Document], n.d. URL https://data.worldbank.org/indicator/SI.POV.GINI?locations=MM (accessed 9.30.22).

[55] Gini index - Chad. | Data [WWW Document], n.d. URL https://data.worldbank.org/indicator/SI.POV.GINI?locations=TD (accessed 9.30.22).

[56] Gini index - Mali. | Data [WWW Document], n.d. URL https://data.worldbank.org/indicator/SI.POV.GINI?locations=ML (accessed 9.30.22).

